# How Term Began at the Hospitals

**Published:** 1909-10-09

**Authors:** 


### Starting Work.

### HOW TERM BEGAN AT THE HOSPITALS.

**Hospital Dinners.**

The annual old students' dinner of St. Bartholomew's Hospital was held in the Great Hall on Friday night last. Dr. Herringham, supported by Lord Sandhurst, Mr. Butlin, F.R.C.S., Sir Clifford Allbutt, the Master of Charterhouse, and the members of the hospital staff, presided. Lord Sandhurst, as treasurer of the hospital, in responding to the principal toast, said that his ten months in office had been quite long enough for him to learn that the relations between the governors and the staff of the hospital were most harmonious; 270 of their old students were serving in the Army and Navy, while about 240 were engaged in medical service in foreign lands. The old students would be glad to know of the success of the new pathological block, which contained all the newest things of the day. This great work had been a burden on the funds of the hospital, which he hoped all those interested would bear in mind.

At St. Thomas's Hospital the annual students' dinner was also held on Friday night, the Hotel Cecil being the foregathering place. Mr. Lawford presided, and Professor Osler was one of the guests of the evening. To the Chairman's toast, "St. Thomas's Hospital and Medical School," Mr. Deputy Harris and Mr. Wallace responded. The former, replying on behalf of the governors, said that although the hospital was an excellent institution, there was room for improvement, and cited in this respect the out-patient department. Last year there were 7,000 in-patients, but, unfortunately, the number of students had not increased proportionately.

At the Trocadero Restaurant the same evening, Mr. John Murray presided over the annual students' dinner of the Middlesex Hospital. Among the guests were Prince Francis of Teck and Major-General Cheylesmore. A most enjoyable evening was spent, and the usual toasts were duly honoured.

The Charing Cross Hospital students' dinner was held at the Criterion on Monday evening last, Sir Clifford Allbutt being the chief guest.

At Guy's, on Thursday night, the students' dinner preceded the opening meeting of the Pupils Physical Society, when Sir Hector Cameron delivered an address on "A Plea for the Treatment of Abscess by Lister's Antiseptic Method." The Westminster students foregathered on Tuesday night at the Imperial Restaurant, Dr. Hebb occupying the chair. Sir George Bartlett, in replying to the principal toast, strongly supported the voluntary principle of hospital support. "I cannot think," he concluded, "of anything worse that could happen to these institutions than to be handed over to the local rates or to Imperial taxation."

---

**St. George's Hospital.**

Here Dr. Rolleston opened the session with an interesting address on "St. George's and the Progress of Physic," in which he discoursed chattily on the medical and surgical worthies who have shed lustre on the western hospital by their names and achievements. There was, for example, Matthew Baillie, whose monument can be seen in the Abbey, and who held office from 1787 to 1800. He was early distinguished by publishing, in 1793, the first book in the English language devoted solely to morbid anatomy, a volume which went through five English editions up to 1818, and was translated into French, German, and Italian. It was of small size compared with modern works on the same subject, and consisted almost entirely of personal observations. Baillie first recognised cirrhosis of the liver, though not under that name, which was invented by Laennec in 1819, as a disease distinct from "scirrhus" tumours; he spoke of it as "common tubercle of the liver." He also first pointed out that "polypi of the heart" were really blood-clots, and drew a distinction between cysts and hydatids of the kidney. This work was subsequently supplemented by "An Atlas of Modern Anatomy" (1799-1802), illustrated from his private collection of morbid specimens which he presented to the Royal College of Physicians in 1819, four years before his death. Baillie's best work was done, as was so commonly the case, before he was forty, for just about this age the demands of private practice, which brought him in £10,000 a year, necessitated his retirement from the hospital, and eventually wore him out at sixty-two, a premature age for his long-lived family.

**Brodie, Jenner, and Young.**

Turning to later lights, Dr. Rolleston gave details of the histories of Thomas Young, Brodie, and Edward Jenner. Young, it seems, combated the infectivity of tuberculosis, and on statistical grounds concluded that the occurrence of conjugal tuberculosis, or infection from husband to wife, or *vice versa*, was not proved. His experiences as a physician were interesting in connection with the question how far the possession of really great abilities or genius bears on the acquisition of worldly success in the practice of his profession. He was popular as a physician and was but little followed in the wards by students, who regarded him as "a great philosopher, but a bad physician." His hospital practice appeared to have had only the negative virtue of being less "meddlesome" than that of his colleagues, and that as a result a greater proportion of his patients were discharged cured from the hospital than of those under the more "vigorous" treatment of the other physicians. But, as Dr. Dickinson said when commenting on Young, the public judged a physician by his methods, not by his results. Brodie's physiological and pathological work entitled him to be included among "The Masters of Medicine." He was the first surgeon, the only other being Lord Lister, to be President of the Royal Society. In this connection it was interesting to note that the only physicians who had occupied the presidential chair of the Royal Society were Sir Hans Sloane (1727-1740), Sir John Pringle (1772-1778), and Wollaston (1820), who, however, threw up this profession in disgust when he was rejected in 1800 as a candidate for the office of physician to St. George's Hospital.

**Jenner's Forgotten Discoveries.**

Dealing with Jenner, Dr. Rolleston said that a discovery of Jenner's, sufficient to make the reputation of any ordinary man, would have been entirely cast into the shade by his other achievements had he published it. It was that angina pectoris depends on disease of the coronary arteries. As was well known, John Hunter suffered from angina pectoris for the extremely long period of twenty years, from 1773 to his death, from that disease, in 1793. In 1776 Jenner saw Hunter, diagnosed his disease, and wrote to the elder Heberden, who was one of Hunter's medical advisers and had recently given a clinical account of the malady, mentioning two cases in which necropsies showed coronary disease. Light was thrown on the pathology of angina pectoris by Sir Benjamin Brodie's observations on intermittent claudication, or limp resulting in muscles supplied by arteries narrowed by disease, the attenuated blood supply being sufficient for the muscles when at rest, but being so inadequate for active contraction that cramp soon supervened.

**An Antarctic Explorer at Middlesex.**

At Middlesex, students were encouraged by Mr. Shackleton, of South Polar reputation, and Dr. Goodall. Mr. Shackleton said they had three doctors with the *Nimrod* expedition and had come through without any deaths (laughter)—but that was due to the fact that they had these medical men with them. As a matter of fact, one man lost an eye down there, and an eminent doctor afterwards said the operation had been performed as satisfactorily as in a London hospital. That was not faint praise when they considered that for the purposes of the operation the patient had been stretched on a board among the ice. A man also lost a big toe through frostbite, and he still carried the toe about in a bottle as a trophy. It was strange that in those cold regions the rotifers should bring forth their young and live in such low temperatures. The temperatures of the explorers had also suffered some curious reverses. On the plateau their temperature was down to 93 or 94—and it was usual to put a white sheet round a man when he was like that—but fortunately their temperature became normal again afterwards. It had been said that people never caught a cold in the Polar regions, and the members of his expedition never did till they opened a bale of clothing which had been packed up in England; the germs were there and they caught cold then, but it disappeared when they went out, while those who remained in the hut still suffered.

**A Long Walk.**

Dr. Goodall gave some statistics and pointed some morals in his address on "Walking the Hospitals." At present, under favourable conditions (such as obtained at Middlesex), the process of walking took about five years, this being the minimum time allowed by law. At other hospitals—and even at Middlesex occasionally—it has been known to take longer—the long distance (time) record at present being held by a gentleman who has up to now devoted twenty-two years of his life to it, without either tiring of the amusement, ruining his health from over-study, or satisfying the examiners! In the case of the London University medical curriculum, five and a half years was the minimum time allowed, the extra half-year having recently been added, possibly to enable the teachers and the students to master the regulations. Recent statistics showed that the average time taken to qualify was six years eleven months, only 13.8 per cent. obtaining their qualification in the *minimum* time, 35.9 per cent. qualifying in the sixth year, and 51 per cent. taking seven years or longer. During these nominal five or five and a half years the student was expected by the examiners to acquire a "sound" knowledge of twenty-eight different subjects; by the teacher of each of these subjects he was expected to obtain an expert knowledge and to devote all his time to it!

**At the Royal Free.**

The opening sessional address on "Pioneering" was delivered at the Institution, Hunter Street, by Mrs. Henry Fawcett, LL.D., under the presidency of her sister, Mrs. Garrett Anderson, M.D. The chief feature of interest during the year has been the consent of the Royal College to admit women to their examinations. Thus, after thirteen years, the efforts of the school have been crowned with success. During the year several meetings of the committee of the University of London appointed to consider the incorporation of the school with the University have been held, and some progress has been made with the scheme. Mrs. Fawcett, in the course of her interesting address, made a brief allusion to Mrs. Anderson's pioneer work in opening the medical profession to women in England, ably seconded as she was by Dr. Sophia Jex Blake, Dr. Edith Pechey Phipson, and others. She dealt at greater length on the pioneer work in the same direction done by another Englishwoman some fifteen years earlier in the United States—Dr. Elizabeth Blackwell. Dr. Blackwell had innumerable difficulties and an indomitable spirit in overcoming them. The students of the present day could hardly have in their hands a more interesting and inspiring book than Dr. Blackwell's autobiography, called "Pioneer Work in Opening the Medical Profession to Women," published by K. Barry, of Hastings.

**Dr. Miers at Mary's.**

At St. Mary's Hospital Medical School the prizes were distributed by Dr. H. A. Miers, Principal of the University of London, who gave an address to the students on the subject of "Theories." The Dean (Mr. W. Clayton-Greene), in presenting the annual report, said that thirty-one beds in the Clarence Wing had now been opened for treatment by therapeutic inoculation. This, following the endowment by Mrs. Henriques of the new operating theatre in the early part of the year, led them to hope that funds would soon be forthcoming to enable the entire wing to be furnished and opened for the reception of patients. The entry of new students for the year promised to equal, if not exceed, that of the last two years.

**Sir John Tweedy at University.**

At University College Hospital the prizes were presented and an address was delivered by Sir John Tweedy, LL.D., F.R.C.S., past President of the Royal College of Surgeons and Emeritus Professor of Ophthalmic Medicine and Surgery in University College. Referring to the death of Dr. Radcliffe Crocker, Sir John said Dr. Crocker had left with them "the memory of an able and amiable fellow worker and the grateful recollection of a long record of much work well done." Sir J. Tweedy then proceeded to congratulate the prize-winners, but warned them not to be content with their scholastic triumphs. For complete success in life it was not enough to be apt in acquiring knowledge or to possess a retentive memory, or to have facility in written or verbal expression. Character was also needed, and all that its term connoted—thoughtfulness, sympathy, courtesy, and culture.

**The London M.D.**

The session was opened on Monday last at the Charing Cross Hospital Medical School. Sir Clifford Allbutt, in the absence of Lord Ridley, distributed the prizes and addressed the students. In presenting his report for the year, the Dean (Mr. F. C. Wallis, F.R.C.S.) drew attention to the question of the London University degree, which has become a matter of vital importance to the London schools. It appears that the public give the preference to medical practitioners having the M.D. degree, and young men entering the profession without it are placed at a great disadvantage, though they may be equally competent. In recent years the superior facilities for obtaining the degree offered by the provincial Universities have exercised a very prejudicial effect upon the metropolitan schools by drawing students away. Former students now in practice, though they would prefer to send their sons to their own school, to which they are affectionately attached, are compelled by the inexorable law of demand to send them elsewhere in search of the M.D. degree. A serious falling-off in numbers has taken place, and one result is that the Conjoint Board of the Royal Colleges of Physicians and Surgeons has had to part with the fine examination hall on the Embankment, of which the foundation stone was laid by Queen Victoria less than 25 years ago. A letter signed by all the medical schools has been sent to the Prime Minister in the sense of the following resolution: "That the Royal Commission, which the Senate of the London University has requested the Government to appoint, should be allowed to take evidence as to the means by which the medical degrees of the University might be made more accessible to London students."

**Lord Justice Moulton at Leeds.**

The winter session at Leeds was opened by Lord Justice Fletcher Moulton on October 1. The Vice-Chancellor of the University (Sir Nathan Bodington), introducing his Lordship to the assembly, paid a warm tribute to his versatility of mind. "In these days of what seems to some of us to be an excessive specialism," continued Sir Nathan, "we ought to have great admiration for a man who has approached so far as is, in any way, practicable to the Baconian ideal of making the whole realm of knowledge his own." Lord Justice Moulton's address dealt with "Some Thoughts on Causation in Health and Disease." At the outset he declared that he knew well the value of special study in any branch of science, and could understand how presumptuous it was for anyone like himself without the specialist's knowledge to address that assembly on such a topic. Still, a layman like himself might find some aspects on the subjects they were studying to which he might with propriety call their attention, for there was this characteristic of therapeutic science: that laymen were interested in its results as much as the professional men. He claimed to have been a close observer of the advance of this science from the time when, at college, he first read the lecture of Lord Lister on the introduction of antiseptics into surgery. Passing to review the 40 or 50 years that had elapsed since the birth of the new conception of the nature and causation of infective diseases, his Lordship alluded to the classical experiments which demonstrated that anthrax was due solely to the presence of a definite micro-organism. From that point one infective disease after another was tracked experimentally to its cause, and in each case it was found that the cause was the invasion of a specific micro-organism. Now one instinctively thought of living germs as the cause of all infective disease, and the instinct had not proved misleading.

